# Idiopathic Hypoparathyroidism and Severe Hypocalcemia in Pregnancy

**DOI:** 10.1155/2018/8316017

**Published:** 2018-11-27

**Authors:** Carine Ghassan Richa, Ali Ihsan Issa, Akram Salim Echtay, Mohamad Souheil El Rawas

**Affiliations:** ^1^Department of Endocrinology, Rafic El Hariri University Hospital, Beirut, Lebanon; ^2^Endocrinology Fellow, Lebanese University, Hadath, Lebanon; ^3^Head of Endocrinology and Internal Medicine Departments, Rafic El Hariri University Hospital, Beirut, Lebanon; ^4^Endocrinologist, Rafic El Hariri University Hospital, Beirut, Lebanon

## Abstract

The objective of this study is to report a case of severe hypocalcemia secondary to hypoparathyroidism in a pregnant woman. We report a case of a 45-year-old woman who presented for tonico-clonic seizure in the third trimester of gestation. She was diagnosed with idiopathic hypoparathyroidism for the first time during pregnancy. She was successfully treated with calcium and calcitriol in the rest of her pregnancy with resolution of symptoms but her infant was born with hypercalcemia and secondary hyperparathyroidism due to the late maternal presentation. To the best of our knowledge, hypoparathyroidism is a disorder rarely observed during pregnancy, resulting in most cases from surgical thyroidectomy.

## 1. Background

Many reports presented different cases of hypoparathyroidism diagnosed before pregnancy, and treatment was adjusted during and postpartum. Cases of idiopathic hypoparathyroidism identified for the first time in pregnancy are rare, especially in the third trimester. Diagnosis and treatment of hypoparathyroidism during pregnancy are challenging. Herein, we describe a rare case of idiopathic hypoparathyroidism with extremely low calcium level and with a late diagnosis affecting fetal outcome.

## 2. Case presentation

A 45-year-old woman G2P0A1 was admitted to the emergency department at 27 weeks of gestation for tonico-clonic seizure. As past medical history, she has a poorly controlled epilepsy diagnosed in childhood, currently on carbamazepine (300 mg twice daily), one unexplained abortion 5 years ago, at 16 weeks of gestation, and gestational diabetes mellitus on metformin (500 mg three times daily) identified 3 weeks prior to presentation. She had recurrent seizure attacks in the past 4 years but this is the first time when it occurs during pregnancy. No calcium level measured prior to the actual admission.

There is no family history of hypoparathyroidism and no surgery of the thyroid or parathyroid glands.

Emergent CT brain revealed no abnormalities. Metabolic workup showed life-threatening hypocalcemia with hyperphosphatemia. Other laboratory data are showed in Table 1.

 1.25 vitamin D was not measured. Neck ultrasound was normal.

After controlling her seizure with the appropriate antiepileptic drugs, she started complaining of paresthesia all over her body, especially in her extremities. Intravenous calcium gluconate (11 grams), magnesium sulfate (2 grams), and levetiracetam (1 gram twice daily) for her seizure were administered and symptoms rapidly resolved and thereafter switched to oral calcium (600 mg 3 tablets every 6 hours) with calcitriol (2 mcg daily) and vitamin D replacement (10 000 IU daily). Based on these results and in front of the hypocalcemia, hyperphosphatemia, and the inappropriately normal PTH, idiopathic hypoparathyroidism associated with vitamin D deficiency was on the top of the differential diagnosis in this patient. No genetic studies performed. Fetal ultrasound was unrevealing. She was discharged home on oral calcium (600 mg 3 tablets every 8 hours), calcitriol (2 mcg daily), oral magnesium and vitamin D supplementation, and antiepileptic drugs with close monitoring of calcium level in order to keep the level in the lower limit of normal. After 4 weeks, caltrate and calcitriol doses were decreased to 600 mg 2 tablets twice daily and 1 mcg daily, respectively, because corrected calcium level increases to 8.8 mg/dl. Variation of calcium level was reported in Table 1. She was followed up clinically and biochemically all her third trimester with a stable calcium level of around 8 mg/dl and without any symptoms.

At 37 weeks of gestation, she presented for delivery, calcium level was 8.5 mg/dl with normal albumin level, and 25-hydroxyvitamin D after 8 weeks of replacement was 20 ng/ml. She gave birth to a baby boy (Apgar score 9/10) with hyperparathyroidism (PTH of 68 pg/ml) and hypercalcemia of 10.7 mg/dl after an uneventful normal vaginal delivery and thereafter she started breastfeeding.

2 weeks postpartum, calcium level increased to 9 mg/dl; therefore calcitriol was given at a dose of 0.5 mcg daily and caltrate at a dose of 1200 mg daily. Oral calcium dose was adjusted every 3 weeks during lactation period, to prevent elevation of calcium level. She had a stable calcium level of around 8.4 mg/dl during the 2 months of lactation. Her baby was followed and monitored for any hypercalcemic side effect by the pediatric team.

## 3. Discussion

Hypoparathyroidism in pregnant women was first described in 1966. There was a conflict in hypoparathyroid women about becoming pregnant [1]. Hypoparathyroidism is rare in pregnancy. It is associated with maternal and fetal complications [2]. Diagnosis during pregnancy is challenging because symptoms are not specific and laboratory findings may be masked by changes in calcium and phosphate homeostasis observed in pregnancy [3].

There is no clear therapeutic regimen for the treatment of hypoparathyroidism during pregnancy [1]. Inadequate management of hypoparathyroidism can lead to miscarriage, stillbirth, preterm labor, and fetal acute respiratory distress syndrome [2]. Parathyroid hyperplasia was found in infants who did not survive [4].

The prevalence of hypoparathyroidism is ranging from 0.5 to 6.6%. There is no available data in pregnant women.

The most common cause of hypoparathyroidism in the population overall is thyroid surgery done mainly for Graves' disease, thyroid carcinoma, or parathyroid surgery. The fact that thyroidectomy is the main cause of hypoparathyroidism, this range depends mainly on the baseline thyroid disease, thyroid volume, the extent of thyroid ablation, and personal experience of a surgeon [3]. Idiopathic hypoparathyroidism, branchial arches disorder (Di Georges syndrome), and autosomal dominant hypocalcemia are among all the other causes [2].

Primary hypoparathyroidism can be part of type I autoimmune polyglandular syndrome, a rare disorder characterised by autoimmune hypoparathyroidism, Addison's disease, and mucocutaneous candidiasis [3]. In our case, these features are not present, and vitamin D deficiency cannot explain the inappropriately normal PTH value, but may demonstrate the severe hypocalcemia, making hypoparathyroidism the most likely diagnosis.

In pregnancy and lactation, there is an increase in calcium demand [5]. Fetus requires 25 to 30 g of calcium for adequate bone formation [4]. 80% of the 30 g of fetal requirement in pregnancy is delivered via the placenta in the third trimester, depending mainly on PTHrP [6]. Maintenance of calcium metabolism during pregnancy and lactation is through increasing in calcium absorption and resorption respectively [5]. In normal pregnancy, total serum calcium decreases because of the increase in maternal blood volume, whereas ionized calcium remains constant [5].

PTH increases mainly in the third trimester because the majority of fetal calcium requirement occurs in this trimester [2]. Normally, in patients with low calcium intake, there is a rise in PTH even in the first trimester [5]. In pregnancy, intestinal calcium, 1,25-dihydroxyvitamin D, urinary calcium excretion, and bone resorption increase [2] 1,25-(OH)2 D3 level rises in the first trimester with a further increase in the third trimester when fetal uptake of calcium reaches its peak. The level then decreases in the third postpartum day [1]. This increase during the second half of pregnancy is mainly secondary to elevated 1a-hydroxylase activity, induced mainly by estrogen, placental lactogen and placental synthesis of calcitriol [7]. During lactation, there are a gradual normalisation of 1,25-dihydroxyvitamin D level, reduced levels of intact PTH, and increased plasma levels of phosphates and ionized calcium [3].

Calcitriol and PTHrP peak at term of delivery; PTHrP is maximal 6 weeks postpartum [8]. Other hormones have contributed to calcium homeostasis in pregnancy like calcitonin, PTHrP, prolactin, placental lactogen, and insulin-like growth factor 1 [2]. PTHrP is the PTH equivalent in fetus. This is demonstrated in animal studies. During early gestation, PTHrP is derived from the placenta and later on from fetal parathyroid glands [1]. PTHrP increased all over the pregnancy, in addition to lactation phase [1].

In hypoparathyroidism, absence of PTH, in addition to increase calcium demand can contribute to hypocalcemia, if untreated; however in lactation, PTH is not required for calcium homeostasis in the presence of PTHrP; thus supplementation can be decreased [2]. Absence of PTH in hypoparathyroidism during pregnancy failed to synthetize active vitamin D [1].

In hypoparathyroid women, the increase in PTHrP during the third trimester was unable to overcome the low PTH; therefore there is an increase in calcitriol requirement especially in the late pregnancy, explaining the delay in diagnosis [1].

Thus, PTH is essential in calcium homeostasis. If deficiency of PTH exists, risk of hypocalcemic complications increases with a wide variety of symptoms including seizure (explaining our case presentation ), cramps, tetany, and heart failure [5]. Hypocalcemia increases uterine irritability, reduces the resting potential, and affects spike frequency of the muscle fibres, which may reveal the spontaneous abortion in this patient during her previous pregnancy [3]. We cannot identify exactly if poorly controlled epilepsy in this pregnant woman was part of undiagnosed hypoparathyroidism, since the diagnosis was made in her childhood, and she had no other manifestations until her current presentation.

Hypoparathyroidism can be ameliorated during pregnancy, reducing calcitriol and calcium doses because of the normal increase in calcitriol and intestinal calcium absorption. However, in certain cases, hypoparathyroidism may worsen due to the inability of the maternal hypoparathyroid status to overcome fetal calcium requirement [6].

Untreated maternal hypoparathyroidism may produce fetal hyperparathyroidism and result in transient hypercalcemia, as a compensatory response to maternal hypocalcemia. In our case, the newborn's hyperparathyroidism was probably secondary to the longstanding untreated severe hypocalcemia, not identified in the mother until her presentation. In turn, overtreatment may induce functional atrophy of fetal parathyroid glands and subsequent hypocalcemia [3]. This case demonstrated that hypoparathyroidism in pregnancy should be correctly treated from the beginning, to prevent any detrimental effect on fetal development.

During delivery, there is an increase in calcium level mediated by prostaglandin E2 that releases body stores [8].

Postpartum, calcium delivery throughout the placenta stopped, stimulating the production of 1,25(OH)2 D3 and the secretion of PTH in the newborn. Synthesis of fetal vitamin D is mediated by maternal kidney, placenta, and fetal kidney. Elevated prolactin, human growth hormone, and estradiol contribute also [1].

During postpartum and lactation, requirement of calcium and calcitriol decreases in all women. This can be explained by elevated PTHrP secreted by the accessory breast glands and prolactin levels which stimulate renal 1-alpha hydroxylase, leading to increase in renal calcium absorption, calcitriol synthesis, and bone turnover and low estradiol which stimulates bone resorption from maternal skeleton [2, 5]. Monitoring of calcium during breastfeeding is required every week [6].

When menstruation is resumed, estrogen level increased leading to decrease in calcium level and subsequent increase in calcium absorption secondary to elevation in calcitriol. Calcium and calcitriol requirements increase after weaning in hypoparathyroid women [8].

Management of hypoparathyroidism during pregnancy is challenging. 50% of undertreated or untreated cases of hypoparathyroidism in pregnancy have serious outcomes [1].

High doses of calcitriol and maternal hypercalcemia have contributed to maternal hypercalciuria, nephrolithiasis, renal impairment, and neonatal seizures in addition to life-threatening complications (such as craniofacial abnormalities, supravalvular aortic stenosis, other fetal malformations, and even teratogenicity) in animal studies, secondary to suppression of fetal parathyroid glands whereas inadequate vitamin D supplementation has led to reactive fetal hyperparathyroidism with intracranial bleed, rickets, skeletal demineralization, subperiostal bone resorption, osteitis fibrosa cystica, and subsequent intrauterine fractures [1, 2, 5]. Thus, hypocalcemia can be life-threatening during pregnancy and adequate doses of vitamin D and its analogs are essential in the treatment [1].

Supplementation by active forms of vitamin D (1,25 (OH)2D3 (calcitriol) and 1a-calcidiol) is preferable because the other forms (cholecalciferol and tachysterol) require liver hydroxylation and have a small therapeutic range with a long half-life, increasing the risk of over and underdosage. Serum calcium should be kept in lower range of normal [1, 5]. Calcitriol has a shorter, dose independent half-life; thus, in case of overtreatment, hypercalcemia will resolve much earlier than with other forms of vitamin D [1]. Other vitamin D preparations have shown a teratogenicity effect [2]. Thus, treatment of choice in hypoparathyroid pregnant women is the combination of oral calcium and calcitriol [1]. Calcitriol is also preferred over administration of intact vitamin D, because the activity of renal 1a-hydroxylase is stimulated by PTH [3]. In this case, our patient did not receive an adequate treatment until her third trimester when she presented and the diagnosis was established. Her calcium level was kept stable in the lower limit of normal in the second half of pregnancy and during lactation, to prevent any fetal harm.

Calcium requirement can reach up to 9g/day during pregnancy in hypoparathyroid women [5].

The risk of toxicity or teratogenicity is minimal as long as serum calcium and 1,25-(OH)2 D3 levels remain in the lower range; this is achieved generally with a dose of calcitriol between 0,25 and 3 mcg/day [1]. If calcium level drops below 1.7 mmol/L, the risks of preterm labor or abortion increase [1]. During pregnancy, recombinant PTH infusion in patients with hypoparathyroidism is not well validated; further studies are needed [5]. Monitoring of calcium level every 3 to 4 weeks during pregnancy and within one week postpartum in the newborn and mother and every 4 to 6 weeks during lactation is required [2].

## 4. Conclusion

To sum up, untreated severe hypocalcemia can lead to life-threatening maternal and fetal complications. Our report presents the case of pregnancy in a young woman with previously unrecognized and untreated hypoparathyroidism, which ended in a delivery of fetus with reactional hyperparathyroidism. Hypoparathyroidism, if effectively diagnosed and treated, cannot be regarded as a contraindication for pregnancy.

## Figures and Tables

**Table 1:** 

	Values	Normal range
Calcium	3.16	8.5-10.5 mg/dl

Albumin	3.2	3.5-5.5 g/dl

Phosphorus	6	2.5-4.5 mg/dl

Magnesium	1.78	1.58-2.55 mg/dl

25-hydroxyvitamin D	<3	20-30 ng/ml

Parathyroid hormone(PTH)	18.31	15-65 pg/ml

Carbamazepine level	0.8	4-12

Thyroid-stimulating hormone	3.1	0.4-4 mIU/L

Spot urine for creatinine	39	28-300 mg/dl

Spot urine for phosphorus	5.28	7-140 mg/dl

Creatinine	0.41	0.5-1.1 mg/dl

## Additional Points

*Learning Points*. (1) Learning how to make diagnosis of idiopathic hypoparathyroidism in a pregnant woman. (2) Understanding the complications of severe hypocalcemia and hypoparathyroidism and be aware of the complications in both fetus and mother. (3) Providing the adequate treatment in such cases with frequent monitoring of calcium level to prevent the detrimental outcomes.

## Supplementary Materials

Supplementary Materials Table 1: changes in calcium concentrations with treatment. Table 2: cases of hypoparathyroidism in pregnancy.
