# Supplementary material for: Idiopathic Hypoparathyroidism and Severe Hypocalcemia in Pregnancy
Source: Case Rep Endocrinol. 2018 Nov 27;2018:8316017. doi: 10.1155/2018/8316017 (PMC6288569; doi:10.1155/2018/8316017)
Supplement: Supplementary Materials — Table 1: changes in calcium concentrations with treatment. Table 2: cases of hypoparathyroidism in pregnancy. [file 8316017.f1.zip › Table 1_CRIE_2587570.pdf]

Table 1: Changes in calcium concentrations with treatment

	Day 1	Day 2	Day 3	Day 4	Day 5	Day 6	Day of discharge	At 31 weeks of GA	At 34 weeks of GA	At 36 weeks of GA	Day of delivery	2 weeks postpartum
Calcium measure												
Total calcium (mg/dl)	3.6	5.66	5.51	5.2	5.4	6	8.04	8.8	7.9	8	8.5	9
Treatment												
Calcium gluconate (g)	11	10	8	6	2	5						
Caltrate (mg/day)		7200	7200	7200	7200	7200	7200	2400	2400	2400	2400	1200
Calcitriol (mcg/day)		2	2	2	2	2	2	1	1	1	1	0.5
