# Supplementary material for: Idiopathic Hypoparathyroidism and Severe Hypocalcemia in Pregnancy
Source: Case Rep Endocrinol. 2018 Nov 27;2018:8316017. doi: 10.1155/2018/8316017 (PMC6288569; doi:10.1155/2018/8316017)
Supplement: Supplementary Materials — Table 1: changes in calcium concentrations with treatment. Table 2: cases of hypoparathyroidism in pregnancy. [file 8316017.f1.zip › TABLE 2_CRIE_2587572.pdf]

Table 2 : Cases of hypoparathyroidism in pregnancy

Authors	Cause of HP[a]	Time of HP diagnosis	Treatment before or at beginning of pregnancy	Treatment during pregnancy	Treatment during lactation	Calcium level range[b]	GA[c]	Maternal and fetal complications
Shah et al(6)	post-thyroidectomy for cold nodules	before pregnancy	2g calcium carbonate<br>400 IU vitamin D3<br>0.25 mcg calcitriol	8g calcium carbonate<br>1600 IU vitamin D3<br>0.5 mcg calcitriol (at 36 weeks of GA)	400mg calcium carbonate<br>200 IU vitamin D3	1.6-1.97	37 weeks	hypocalcemic symptoms during delivery.<br>healthy baby.
Salle et al(4)	idiopathic HP	at 4 weeks of GA	NA[d]	1g calcium<br>1-2 mcg calcitriol	NA	1.72-2.4	37 weeks	CHF[e]+MR[f] at 27 weeks.<br>Twin babies:<br>1-bradycardia<br>2-neurological complications.
Krysiak et al(3)	post-thyroidectomy for cold nodules	at 20 weeks of GA	NA	3g calcium carbonate<br>0.75 mcg calcitriol	3g calcium carbonate<br>0.5 mcg calcitriol	1.4-2.3	38 weeks	healthy baby.
Hatswell et al(2)	idiopathic HP	before pregnancy	1 mcg calcitriol<br>1200 mg calcium carbonate	1.75 mcg calcitriol (decreased to 0.75 mcg in the third trimester)	1 mcg calcitriol	2.13-2.38	37 weeks	healthy baby.
Hatswell et al(2)	idiopathic HP	before pregnancy	2400 mg caltrate<br>0.75 mcg calcitriol	600 mg caltrate<br>1 mcg calcitriol (in the third trimester)	NA	2.17-2.46	41 weeks	healthy baby.
Hatswell et al(2)	AD[g] branchial arch disorder	before pregnancy	1500mg calcium carbonate<br>0.25 mcg calcitriol	2400mg calcium<br>1 mcg calcitriol	NA	2.2-2.94	37 weeks	SGA[h] baby.
Present Case	idiopathic HP	at 27 weeks of GA	7200 mg caltrate<br>2 mcg calcitriol<br>10000 IU vitamin D3	2400 mg caltrate<br>1 mcg calcitriol (at 31 weeks)	1200 mg caltrate<br>0.5 mcg calcitriol	0.79-2.2	37 weeks	baby with secondary hyperparathyroidism

[a] HP: hypoparathyoidim [b] calcium level in mmol/L [c] GA: gestational age [d] NA: not available [e] CHF: congestive heart failure
[f] MR: mitral regurgitation [g] AD: autosomal dominant [h] SGA: small gestational age
